# Biofilm accumulation and sucrose rinse modulate calcium and fluoride bioavailability in the saliva of children with early childhood caries

**DOI:** 10.1038/s41598-022-14583-2

**Published:** 2022-06-18

**Authors:** Camila Lopes Crescente, Emerson Tavares de Sousa, Aline Tavares Lima-Holanda, Carolina Steiner-Oliveira, Marinês Nobre-dos-Santos

**Affiliations:** Department of Health Sciences and Pediatric Dentistry, Piracicaba Dental School, University of Campinas-UNICAMP, Limeira Avenue 901, Piracicaba, SP CEP 13414-903 Brazil

**Keywords:** Dental diseases, Oral diseases

## Abstract

This study aimed at investigating the combined effect of biofilm accumulation and 20% sucrose rinse on the modulation of calcium (Ca^2+^), phosphate (P_i_), and fluoride (F^−^) bioavailability in the saliva of children with early childhood caries (ECC). Fifty-six preschoolers of both genders were evaluated according to caries experience and activity: caries-free (CF, n = 28) and with ECC (n = 28) and then, submitted to biofilm intervention (biofilm accumulation). In each situation, saliva samples were collected before and five minutes after a 20% sucrose rinse to determine the concentrations of Ca^2+^, P_i_, and F^−^. Calcium concentration was significantly lower in the biofilm accumulation situation compared to the situation of biofilm mechanical control (p ≤ 0.01), except for CF children after sucrose rinse. Biofilm accumulation increased salivary calcium concentration in children with ECC after sucrose rinse (p = 0.04), whereas mechanical biofilm control reduced it in both groups (p = 0.000). Phosphate concentration was influenced by mechanical control of biofilm in CF children (p = 0.03). The fluoride bioavailability was reduced by sucrose rinse and biofilm accumulation in CF and ECC children (p ≤ 0.002). In conclusion, the combined effect of biofilm accumulation and sucrose rinse modifies the bioavailability of calcium and fluoride in the saliva of children with early childhood caries.

## Introduction

Early childhood caries (ECC) is one of the most prevalent chronic diseases in children worldwide, affecting children’s quality of life and generating a potential source of cost for their parents and society^1^. One of the main factors for the onset and progression of tooth decay is the presence of biofilm, which is significantly impaired by the sugar bioavailability in the oral environment^2^. Dental biofilm is an organized structure composed of commensal bacteria surrounded by an extracellular matrix that accumulates over the dental structure due to poor oral hygiene^3^. Biofilm function is determined by the composition and properties of saliva, as well as by the dominant nutritional source in the child's diet. In this sense, a diet rich in fermentable carbohydrates such as sucrose provides a coercive environment for a highly specialized oral ecosystem with a predominance of saccharolytic species, with important changes in the physicochemical properties of saliva^4^.

Saliva plays an important role in maintaining the integrity of dental tissues due to the presence of Ca^2+^, P_i_, F^−^ and other inorganic ions. These chemical components can facilitate the remineralization of early caries lesions^5,6^ and promote a supersaturation of minerals, acting strategically to mitigate the damage caused by saccharolytic bacteria^7,8^. Thus, Ca^2+^, P_i_, and F^−^ levels in saliva form a natural defense mechanism against tooth enamel dissolution^9,10^. As has been demonstrated recently^11^, sucrose rinse has a significant effect on the electrolytic concentration of P_i_ and F^−^ in saliva when children with ECC were compared with CF children. In addition, saliva's ability to neutralize the oral environment was different when children with ECC were compared with CF children, and this saliva potential was influenced by both sucrose exposure and biofilm accumulation^12^. This result may reflect the transient shift of the oral ecosystem after sucrose exposure and can be more deleterious for pH-modifying ability in children with ECC. Thus, considering the influence of salivary pH and buffering capacity on the electrolytic dynamic in the oral cavity, it was hypothesized that biochemical changes in saliva can be expected as a consequence of cariogenic challenge and biofilm accumulation.

The most intriguing question here is: Can ECC as a chronic disease influence these electrolytes and explain the boundaries between a healthy and sick oral ecosystem? In this sense, a recent study^13^ demonstrated that the assessment of children based only on the salivary biochemical electrolytes outperformed the prediction status of children with ECC when compared with classifiers based only on the salivary microbiome or on both properties of saliva (microbial and biochemical electrolytes data). That is, assessment of the caries prediction status in children was more reliable when based on the inorganic composition of saliva^13^. Focusing on the bioavailability of the most important ions involved in demineralization and remineralization events (Ca^2+^, P_i_, and F^−^), we intended to conduct research that brings some light and caveats to this question.

This research was designed to improve the understanding the behavior of Ca^2+^, P_i_, and F under two main circumstances involved in ECC physiopathology: sucrose exposure and biofilm accumulation. Previous studies have demonstrated that clinically visible biofilm and a high daily sugar exposure are considered risk factors for ECC^14,15^. Children who present a clinically visible biofilm and are expose to sugar more than 3 times a day are more prone to develop ECC^14^. It is important to emphasize that, the accumulation of clinically visible biofilm is not only related to oral hygiene status but more importantly reflects the sucrose consumption. As recently highlighted by the IAPD Bangkok Declaration^16^, the development of research regarding the prevention and comprehensive management of ECC should be the basis to improve the scope and level of evidence in this field. In this context, results obtained may reinforce the need to establish preventive strategies based on biofilm control and reduced sugar exposure.

Thus, this study aimed to investigate the combined effect of biofilm accumulation and sucrose rinse on the modulation of Ca^2+^, P_i_, and F^−^ bioavailability in the saliva of children with ECC. Our null hypothesis was that after a sucrose rinse, biofilm accumulation would not modify the bioavailability of Ca^2+^, P_i_, and F^−^ minerals differently in children with ECC as compared to caries-free children. Of note, this paper is an extension of a previously published study^12^.

## Results

Fifty-six volunteers finished the study, twenty-eight per group. For the ECC group, the median decay-missing-filled surfaces (dmfs) was 7.00 (Interquartile range: 10.00). The decomposed caries index showed 60% of white spot lesion, 20% of active cavitated lesion, 11% of filled surface without decay, 7% of inactive cavitated lesion, and 2% of filled surface with inactive caries lesion. The sex ratio M–F of the sample was 1.15:1.00 for the CF group and 1.00:1.00 for the ECC group—Table 1.

**Table 1:** Sample characteristics of volunteers.

	CF	ECC	α (effect size)
DMF-S plus ACL^a^—median (IQR)	0	7.00 (10.00)	
Sex (M:F)	1.15:1.00	1.00:1.00	0.79
Total sugar amount (g)—mean (SD)	287.25 (41.16)	306.50 (30.80)	0.026 (0.52)
Total sugar frequency (times/day)—mean (SD)	5.18 (1.55)	5.86 (1.08)	0.032 (0.50)
Percentage of sugar in diet (%)^b^—mean (SD)	53.46 (3.79)	55.09 (3.27)	0.046 (0.45)

Continuous outcomes were tested using independent t-test for α and Hedges’ g for effect size. Sex distribution across the groups was tested using Chi-squared. ^a^The decomposed index was: 60% of White Spot Lesion, 20% of active cavitated lesion, 11% of filled without decay, 7% of chronic cavitated lesion, and 2% of filled with chronic lesion. ^b^The calculation was based on the total sugar amount in relation to all macronutrients from the diet. *DMF-S* number of decayed, missing, or filled surfaces, *ACL* active caries lesions.

**Figure 1:**
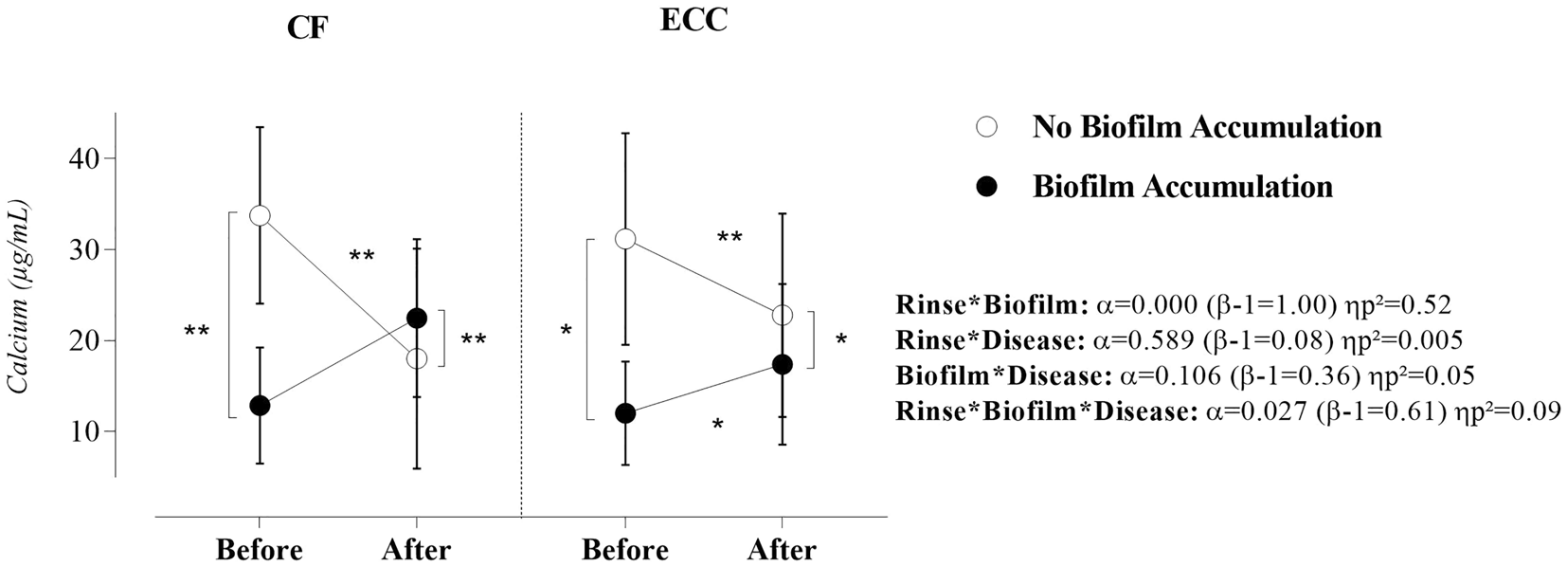
Effect of sucrose rinse, biofilm accumulation, and ECC on the ionic concentration of calcium. Statistical analysis was performed with a sample of 56 volunteers, 28 per group. ηp^2^: Partial eta squared. Data were plotted as means and standard deviations. When significant interactions were found, the main effects were suppressed. A single asterisk represents a significant p-value ≤ 0.05. A double asterisk represents a p-value ≤ 0.01.

As demonstrated in Fig. 1, calcium concentration was significantly influenced by sucrose rinse, biofilm, and ECC (α = 0.027; β − 1 = 0.61; ηp^2^ = 0.09). However, there was no significant interaction between ECC and sucrose rinse (α = 0.589; β − 1 = 0.08; ηp^2^ = 0.005), as well as between ECC and biofilm (α = 0.106; β − 1 = 0.36; ηp^2^ = 0.05). More importantly, it was noticed that the combined effect of sucrose rinse and biofilm mobilized the calcium dynamic in the oral cavity (α = 0.000; β − 1 = 1.00; ηp^2^ = 0.52). In Table 2, simple effects revealed that calcium concentration was significantly lower in the biofilm accumulation situation as compared with the mechanical control of biofilm situation (p ≤ 0.01), except for CF children after sucrose rinse—which was higher than in the biofilm accumulation situation (α = 0.000; β − 1 = 0.99; ηp^2^ = 0.265). In addition, it was evidenced that biofilm accumulation significantly increased salivary calcium concentration in children with ECC after sucrose rinse (α = 0.04; β − 1 = 0.54; ηp^2^ = 0.075) whereas mechanical control of biofilm reduced it in both groups (p ≤ 0.000). The strength of the mechanical control of biofilm can be demonstrated considering the high values of partial eta squared (ηp^2^ for CF = 0.574 and ηp^2^ for ECC = 0.531). Differences between CF children and children with ECC can be observed after sucrose rinse when the mechanical control of biofilm was absent (α = 0.034; β − 1 = 0.57; ηp^2^ = 0.081).

**Table 2:** Simple effects of sucrose rinse, biofilm accumulation, and ECC on the ionic concentration of calcium, phosphate, and fluoride in saliva.

Simple effects		Calcium			Phosphate			Fluoride		
		α	(β − 1)	ηp^2^	α	(β − 1)	ηp^2^	α	(β − 1)	ηp^2^
Biofilm accumulation in CF children	Pre-rinse	**0.000**	**0.998**	**0.315**	0.675	0.070	0.003	**0.000**	**0.984**	**0.251**
	Post-rinse	**0.000**	**0.991**	**0.265**	0.362	0.147	0.015	0.464	0.112	0.010
Biofilm accumulation in ECC children	Pre-rinse	**0.011**	**0.740**	**0.115**	0.648	0.074	0.004	0.390	0.136	0.014
	Post-rinse	**0.017**	**0.677**	**0.101**	0.585	0.084	0.006	0.072	0.438	0.061
Rinse in CF children	No Biofilm accumulation	**0.000**	**1.000**	**0.574**	**0.033**	**0.577**	**0.082**	**0.000**	**1.000**	**0.451**
	Biofilm accumulation	0.089	0.397	0.053	0.449	0.116	0.011	**0.002**	**0.904**	**0.175**
Rinse in ECC children	No Biofilm accumulation	**0.000**	**1.000**	**0.531**	0.107	0.364	0.047	**0.000**	**0.987**	**0.258**
	Biofilm accumulation	**0.041**	**0.540**	**0.075**	0.194	0.252	0.031	**0.000**	**1.000**	**0.363**
CF vs ECC before sucrose rinse	No Biofilm accumulation	0.369	0.144	0.015	0.269	0.196	0.023	0.189	0.258	0.033
	Biofilm accumulation	0.131	0.325	0.042	0.888	0.052	0.000	0.075	0.430	0.060
CF vs ECC after sucrose rinse	No Biofilm accumulation	0.602	0.081	0.000	0.591	0.083	0.005	0.588	0.083	0.006
	Biofilm accumulation	**0.034**	**0.572**	**0.081**	0.434	0.121	0.011	0.599	0.081	0.005

Significant values are in bold.

**Figure 2:**
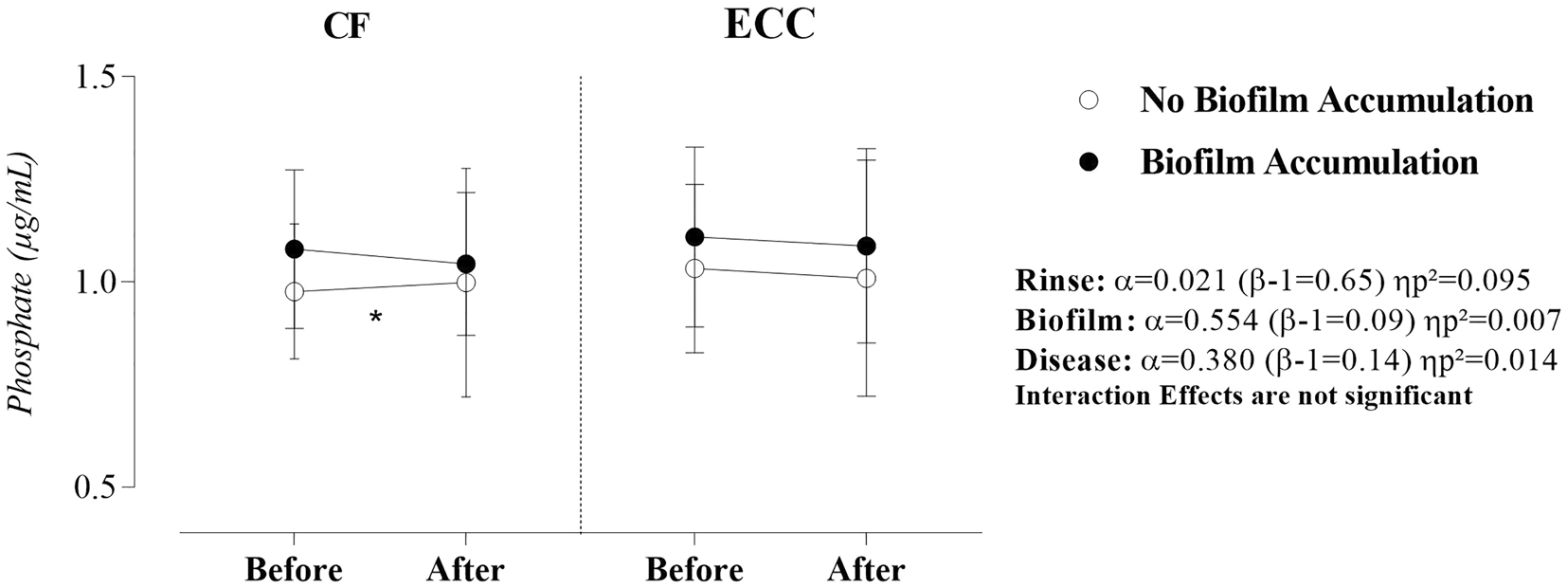
Effect of sucrose rinse, biofilm accumulation, and ECC on the ionic concentration of inorganic phosphate. Statistical analysis was performed with a sample of 56 volunteers, 28 per group. ηp^2^: Partial eta squared. Data were plotted as means and standard deviations. When significant interactions were found, the main effects were suppressed. A single asterisk represents a significant p-value ≤ 0.05. A double asterisk represents a p-value ≤ 0.01.

Concerning phosphate concentration (Fig. 2), there was no significant interaction between sucrose rinse, biofilm, and caries disease (α = 0.479; β − 1 = 0.11; ηp^2^ = 0.009). The main effects evidenced that only biofilm accumulation influenced phosphate concentration in saliva (α = 0.021; β − 1 = 0.65; ηp^2^ = 0.095). Simple effects (Table 2) indicate that this influence was significant for the difference between pre- and post-rinse in CF children when the mechanical control of biofilm was performed (α = 0.033; β − 1 = 0.577; ηp^2^ = 0.082). More specifically, a higher phosphate concentration in saliva was observed after sucrose rinse when CF children were submitted to mechanical control of biofilm.

**Figure 3:**
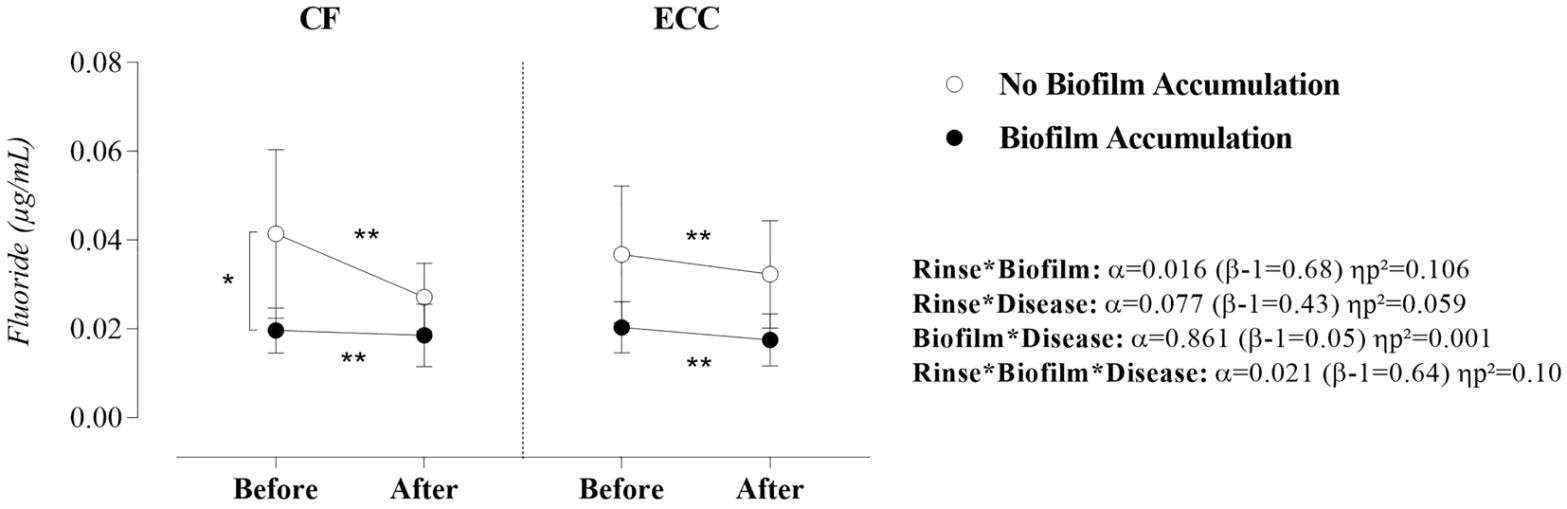
Effect of sucrose rinse, biofilm accumulation, and ECC on the ionic concentration of fluoride. Statistical analysis was performed with a sample of 56 volunteers, 28 per group. ηp^2^: Partial eta squared. Data were plotted as means and standard deviations. When significant interactions were found, the main effects were suppressed. A single asterisk represents a significant p-value ≤ 0.05. A double asterisk represents a p-value ≤ 0.01.

In Fig. 3 it is possible to observe that fluoride concentration was significantly altered by sucrose rinse, biofilm, and caries disease (α = 0.021; β − 1 = 0.64; ηp^2^ = 0.10). Nevertheless, ECC alone did not influence fluoride concentration when considering within-subjects factors such as biofilm accumulation (α = 0.861; β − 1 = 0.05; ηp^2^ = 0.001) and sucrose rinse (α = 0.077; β − 1 = 0.43; ηp^2^ = 0.059). Of note, the interaction effect between sucrose rinse and biofilm was significant (α = 0.016; β − 1 = 0.68; ηp^2^ = 0.106). Simple effects (Table 2) show that biofilm accumulation reduced the fluoride bioavailability in the saliva of CF children in the pre-rinse situation (α = 0.000; β − 1 = 0.98; ηp^2^ = 0.251). Sucrose rinse reduced fluoride concentration in the mechanical control of biofilm and in the biofilm accumulation situations in CF and ECC children (p ≤ 0.002).

## Discussion

This study rejects the null hypothesis because sucrose rinse and biofilm accumulation modifed the bioavailability of Ca^2+^ and F^−^ minerals differently in children with ECC compared to caries-free children. However, the null hypothesis for P_i_ mineral was accepted since there are no interaction effect between sucrose rinse and biofilm accumulation (α = 0.519; β − 1 = 0.098; ηp^2^ = 0.008).

The study design included a simulation of a cariogenic challenge with a sugar concentration similar to sweetened foods and liquids commonly ingested by children plus the simulation of proper oral hygiene supervised by an adult (situation in which there is biofilm control) and deficient oral hygiene (in which there is biofilm accumulation). To ensure sample homogeneity and make the factors that directly influence caries disease more noticeable, some precautions were taken in the eligibility phase, such as selection of preschool children with a well-defined age group (4 to 5 years) to avoid the effect of saliva and microbiome maturation, with access to similar fluoride sources (1.100 ppm fluoride toothpaste and 0.7 ppm fluoridated water) and sharing the same 5 meals a day.

Interestingly, the frequency of sucrose consumption was around 5 times a day for CF children as well as for children with ECC (Table 1), which is considered a sugar ingestion risk for ECC^17,18^. In line with this assumption, in the group of children with ECC, it was observed that 80% of caries lesions were in the active situation (60% active white spot lesions and 20% active cavitated lesions). This critical situation in individuals having early childhood caries inspired us to investigate the salivary factors associated with the physiopathology of dental caries in this group of children and to provide meaningful information to trigger more specific and efficient diagnosis, prevention, and care for this disease.

This study was the first to demonstrate that the concentrations of calcium and fluoride in the saliva of preschool children were significantly influenced by transient exposure to sucrose (rinse) and by biofilm accumulation. Moreover, the high values of partial eta squared in these interactions can evidence the strength of this combined effect (see partial eta squared in Figs. 1 and 3). On the other hand, in the children who experienced ECC, the calcium and fluoride concentrations did not behave differently from CF children in the experimental conditions of the study. This result strengthens the findings of a previous research^11^ demonstrating that the status of the disease (presence or absence) did not influence the bioavailability of naturally occurring salivary electrolytes such as calcium and fluoride. In addition, these results, add more information to the bioavailability of these ions in the saliva medium. Thus, this emerging body of knowledge can be relevant for future experimental designs about ECC physiopathology, which must ensure that proximal factors (simple sugars intake and oral hygiene) should be considered or isolated to explain what changes in the oral environment could be expected in each situation and how these changes could be controlled to avoid more aggressive forms of ECC.

Calcium concentration was significantly lower in the biofilm accumulation situation as compared with the mechanical control of the biofilm situation in both CF children and children with ECC. In the biofilm accumulation situation, these results can be explained by the metabolic profile (more saccharolytic) of the oral environment, which can promote an important variation on salivary parameters such as flow rate, pH, and buffering capacity^12^. Thus, the frequent pH fall may disrupt the equilibrium of calcium ions on the enamel surface and provide a coercive scenario for uptake of calcium ions from saliva. Moreover, there is a possibility of salivary calcium being complexed by acids, mainly lactate^19^, reducing its saliva bioavailability.

There was a tendency to increased salivary calcium after sucrose rinse in the biofilm accumulation situation, whereas a decrease in this ion concentration was noticed in the mechanical control of biofilm situation. As an exception, it was observed that sucrose rinse did not modify calcium concentration in the saliva of CF children in the biofilm accumulation situation. This should be interpreted with caution since the alpha value was in the margin of significance (p = 0.089) and the effect size (type II error) and the strength (partial eta squared) were low. The opposite tendency for calcium concentration in the biofilm accumulation and mechanical control of biofilm situation can be a result of a more organized biofilm with an extracellular polymeric matrix capable of controlling ionic and molecular transport across the biofilm structure^20,21^. As recently demonstrated^19^, cariogenic bacteria species have a regulatory mechanism to eliminate possibly toxic amounts of calcium ions, binding to them to strengthen their advanced biofilm nuclei, and at the same time, affecting the free calcium ratios for remineralization that would be available in the biofilm fluid. In other words, the less organized biofilm structure provides a more meaningful calcium uptake from saliva to the tooth-enamel interface, reducing its bioavailability in saliva, whereas a more organized biofilm does not. Thus, as the organized biofilm can be a barrier to ionic transport, the effect of masticatory and gustatory stimuli on the increase in calcium concentration can be more evident, especially in the group of children with ECC.

When the observed ion is the inorganic phosphate, the analysis of simple effects showed that there was a medium and significant effect of sucrose rinse on CF children in the situation of mechanical control of biofilm. A different result was observed in a similar research^11^ which demonstrated that CF children maintain their inorganic phosphate concentration stable after the cariogenic challenge with sucrose rinse. A deeper theoretical consideration can be inferred from these contrasting data because, in CF children more than ECC alone, they reinforce that the mechanical control of biofilm rules the response to the transient exposure to sucrose. In this context, only biofilm accumulation influenced phosphate concentration in saliva in the experimental conditions of our study. On the other hand, previous clinical studies demonstrated that phosphate concentration is inversely related to the dental caries experience^22,23^. The contrasting results may well be explained considering the differences in the design of the various studies. In one study^22^, authors used unstimulated submandibular and sublingual saliva samples obtained from adolescents aged 13–15 years old, who had a mean DMFS of 25.9 (SD ± 8.5). In another investigation^23^, the experimental group was composed of caries-active children aged 4 to 8 who presented a DMFS ≥ 5. Furthermore, it should be perceived that in none of these referred studies were the individuals exposed to a cariogenic challenge and/or refrained from controlling their biofilm.

As expected, in both groups, sucrose rinse reduced fluoride concentration in the mechanical control of biofilm and biofilm accumulation situations. Thus, our research reinforces the overwhelming influence of sucrose rinse and biofilm on the behavior of this ion when compared with the caries experience. However, it should be noted that although the interaction between sucrose rinse and disease was not significant, the p-value of the interaction between the two variables was 0.07 with low power but medium partial eta squared (see Fig. 3). This may indicate that the difference exists but the sample size did not make it possible to demonstrate this effect. More specifically, biofilm accumulation caused an impact on fluoride bioavailability in the pre-rinse situation in CF children (α = 0.000; β − 1 = 0.984; ηp^2^ = 0.251) and probably in the post-rinse situation in ECC children (α = 0.072; β − 1 = 0.438; ηp^2^ = 0.061), if the power of the analysis increases. Here, the number of participants poses some restrictions to statistical inference and design. In this respect, we argue that the three-way analysis of variance has a complex statistical structure and limited studies are available with similar factors investigated together to assess the expected variability and appropriate sample size.

Fluoride concentrations in saliva may be related to fluoride concentrations in the biofilm/tooth interface, which is the site of action for the fluoride effect on caries control. There is a consensus that biofilm gradually concentrates the fluoride available from saliva, enabling higher levels of F^−^ ions available on the tooth surface^24^, which can explain why the presence of biofilm causes a reduction in the levels of available fluoride in saliva in CF children. However, the higher levels of salivary fluoride in CF children when mechanical control of biofilm was performed, could represent a state of more stability against tooth demineralization since more ionic components would be available for crossing the disorganized biofilm structure, reducing the chemical aggression promoted by the cariogenic challenge. Thus, biochemical processes in the oral cavity can increase the environmental electrolytes supersaturation around and within the tooth surface biofilm structure^25^, which can be highly indicative of oral health status.

Several strengths can be pointed out in this research, such as the careful selection of an adequate and specific sample, the collection of data simulating usual day-to-day conditions, and the statistical design with a robust and extremely conservative rank test of interaction. However, some limitations should be displayed. Firstly, we only analyzed saliva samples and did not verify the bioavailability and behavior of the inorganic composition in biofilm samples, which could provide us with important results. Secondly, there are no longitudinal data to inform that the bioavailability of ions in saliva can be changed over time, considering the ECC outcome. This limitation includes knowledge regarding the boundaries between children with ECC whose disease activity is arrested or maintained and CF children who develop ECC or maintain their health status. Thus, future investigation considering a longitudinal design is highly recommended to address these aspects of ECC. Thirdly, the design of this research considered the caries experience and activity as a parameter for classifying children with ECC. In this sense, the absence of stratification of ECC components in an experimental design can be a potential source of bias.

In conclusion, this study demonstrated that the combined effect of biofilm accumulation and sucrose rinse modifies the bioavailability of calcium and fluoride in the saliva of caries-free children and children with ECC. On the other hand, for phosphate concentration, only sucrose rinse seems to be relevant for caries-free children in the absence of biofilm accumulation.

## Methods

### Research subjects

The sample size was calculated using Gpower 3.1 software, assuming a difference between two independent means. The calculation was performed considering an α value of 0.05, β value of 0.10, allocation rate of 1/1, and confidence interval of 0.95. The sample size was estimated according to the mean and standard deviation (SD) of salivary calcium concentration in the saliva of children with ECC (18.49, SD 6.6) and caries-free children (28.16, SD 12.8)^26^, resulting in 25 subjects for each group. The calculated number (25) was increased to 30 to compensate for withdrawals during the experiment. Thus, 30 preschoolers per group were included in this study.

The sampling plan was performed using random single-step clustering method. From a total of 89 public daycare centers in the city of Piracicaba, São Paulo, Brazil, a cluster of three public daycare centers were selected and children were analyzed and included in the study according to the eligibility criteria. Children with caries lesions (dmfs ≥ 1) and caries-free (dmfs = 0), aged 3 to 4 years (at the beginning of the study) and of both sexes were included in the study. Preschool children whose parents or guardians refused to participate in the research or who did not cooperate with the clinical examinations were excluded from the research, as well as those who, in the clinical examination, presented periodontal disease (assessed using the Gingival Bleeding Index^27^), severe fluorosis or who were using any orthodontic appliance. In addition, children who had any systemic disease, motor neuron or communication difficulties, and who were under antibiotic therapy were excluded. This information was accessed through a questionnaire sent to parents.

### Experimental design

An experimental and parallel study was carried out with a sample consisting of children with ECC and caries-free children, to investigate the influence of two experimental conditions on the inorganic composition of saliva: biofilm accumulation and cariogenic challenge with a 20% sucrose solution.

**Figure 4:**
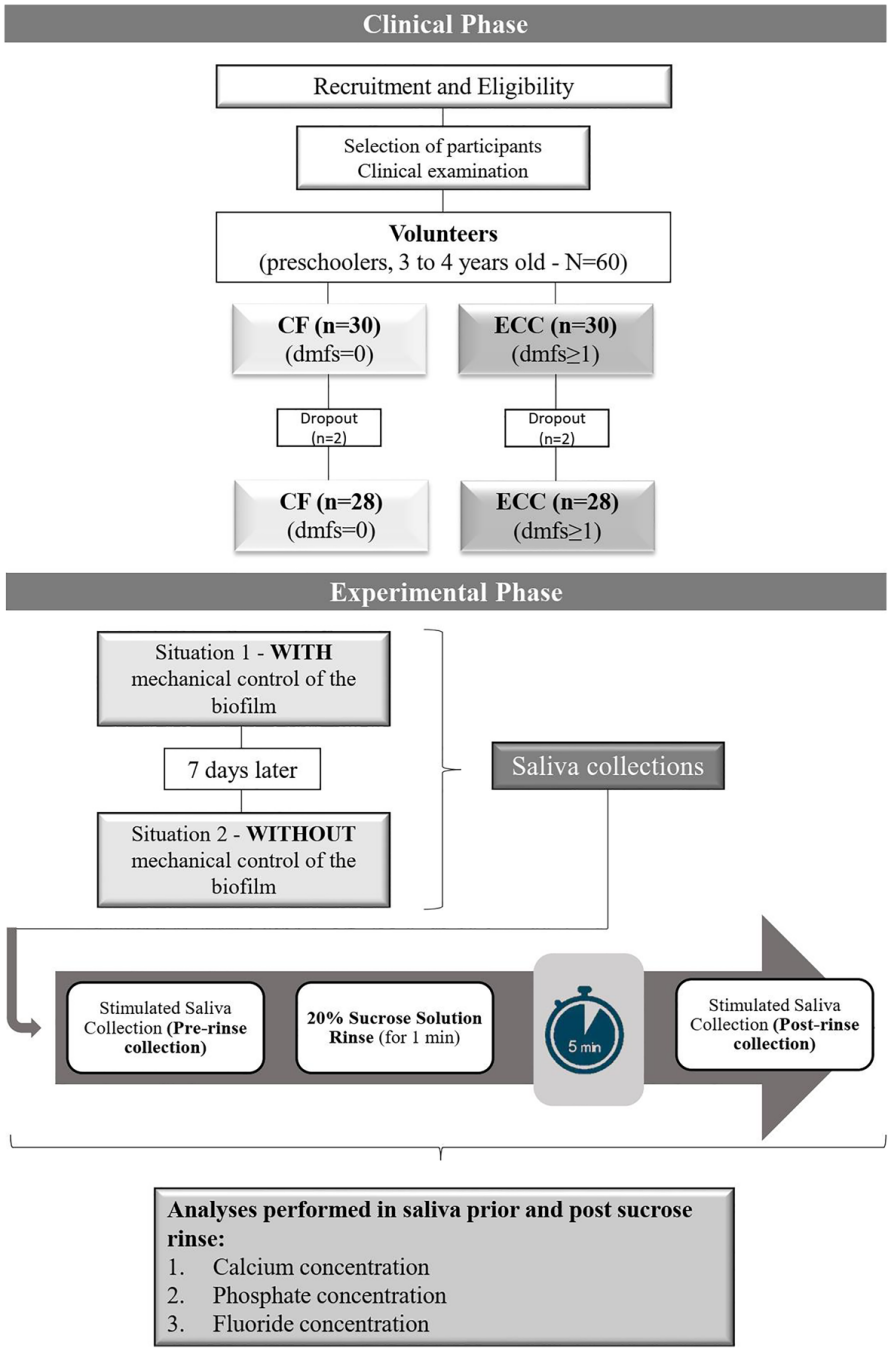
Flowchart of the research experimental design.

Adequate oral hygiene (situation of biofilm control) and poor oral hygiene (no biofilm control) was performed at different times with an interval of at least one week. These interventions were conducted as follows (Fig. 4):

- *Absence of biofilm accumulation* For two days, the volunteers were submitted to mechanical control at least three times a day with a technique suitable for the preschool age group (Fones technique). Tooth brushing was performed 1 h before saliva collection and always supervised by the researcher in the morning (between 8 and 9 am) and in the afternoon (between 4 and 5 pm). Phone calls and/or text messages were used as a strategy to remind parents about nighttime toothbrushing.
- *Biofilm accumulation* For two days the parents were instructed, through phone calls and/or text messages, to abstain from brushing their children's teeth. To strengthen this recommendation, the children were also instructed about the procedure. Visualization of the visible biofilm on the upper incisors^28^ was used as a control for abstaining from brushing.

In each situation, samples of stimulated saliva were collected before rinsing for 1 min with 10 mL of a 20% sucrose solution and after five minutes. The saliva samples were used to determine the inorganic composition of Ca^2+^, P_i_, and F^−^. Information regarding dietary habits was collected considering the use of three-day diet diary.

### Clinical examination

The clinical exam aimed to provide information regarding the dental caries status. The examination was performed at the daycare center, by a single previously calibrated dentist. The examiner was trained in a population of the same age as the sample. The training exercise consisted of two steps (theoretical and clinical). The first stage involved a theoretical discussion of the World Health Organization (WHO) caries diagnostic criteria, plus the criteria for the diagnosis of active caries lesions^29^. The WHO and active caries lesions criteria and codes were adopted following a previously validated protocol^30^. The second step was carried out to assess the consistency of the clinical analysis. The examiner (ETS) and a calibrated pediatric dentist (CEO) evaluated the indices in a sample of 10 children, selected at random. The inter-examiner reliability was verified using the Kappa statistic (κ = 0.89) and the reproducibility of the diagnosis was determined by the intra-examiner Kappa index based on reassessments performed in 60% of the children after a one-week interval (κ = 0.95).

During the examination, individual protective equipment was used, as well as sterilized and individual clinical material for each child. For the examination, a portable flashlight with LED light (Sfl5540, Wat Nichia Philips, São Paulo, SP, Brazil), a clinical mirror, and a blunt tip explorer (Indusbello, Londrina-PR, Brazil) and/or sterilized gauzes (Medi House, São Paulo, SP, Brazil) were used. Portable dental equipment with a triple syringe (Odontocase Basic Line, Rio de Janeiro, RJ, Brazil) was used to facilitate the visualization and correct diagnosis of the initial caries lesions (active white spot lesion).

Children who needed dental treatment were referred to the pediatric dentistry clinic of the Piracicaba Dental School—UNICAMP, where they received comprehensive care.

### Analysis of dietary habits

Parents or guardians filled out a three-day diet diary. The diary specified the children's eating habits, including the time of feeding and the amount and content of all meals and snacks. A detailed review of all food items and meals throughout the day was performed to check for errors or omissions. More detailed information was collected during an interview with the children’s parents or guardians. This information included food type, brands, food preparations, bottle use, bottle preparation, and nighttime breastfeeding. In addition, considering that all children included in this research stayed at the daycare center full time (from 7:30 a.m. to 5:00 p.m.), the content of the institutional menu was collected and the information was added to the analysis. The daily frequency of sugar exposure was counted and then divided by the number of days (three). A blinded nutritionist analyzed the collected dietary information and using dietary analysis software Dietbox (Dietbox for Windows, Porto Alegre, RS, Brazil) assessed the amount of fermentable carbohydrates ingested by each child. The amount of sugar ingested per day (grams/day) and the relative percentage of sugar amount with relation to all macronutrients from the diet were estimated by the average consumption in grams in the three days of dairy filling.

### Saliva collection

Stimulated saliva collection was performed with a piece of Parafilm M (Pechiney Plastic Packaging, Manuf. and Markets Plastic, Chicago, IL, USA). The saliva produced in the initial 30 s was discarded and the remaining saliva was collected for 5 min in autoclaved graduated tubes. After collection, the saliva samples were centrifuged at 16,097.2×*g* for 15 min and then transferred for 2.0 mL eppendorf identified microtubes and kept frozen at – 40 °C until the analyses were performed.

### Calcium analysis

Saliva calcium concentration was analyzed by the direct colorimetric method using a microplate spectrophotometer (PowerWave HT, BioTek Instruments, Winooski, VT, USA). Briefly, 25 µL of saliva were pipetted into a 96-well plate to react with 125 µL of calcium-sensitive reagent (Arsenazo III). The reader was precalibrated against a calcium carbonate standard curve (0–100 µg/mL) and readings were performed using an λ 650 nm of absorbance^31^. The ionic concentration of calcium in saliva was obtained from the absorbance values using a linear equation (y = ax + b) in a curve fit above 0.98. Samples were analyzed in duplicate. Calcium concentrations were expressed as µg/mL.

### Phosphate analysis

Phosphate concentration in saliva was analyzed by the colorimetric method using a microplate spectrophotometer (PowerWave HT, BioTek Instruments, Winooski, VT, USA). Briefly, 25 µL of saliva were pipetted into a 24-well plate to react with the phosphate reducing agents (molybdic acid and alpha-aminonaphthol sulfonic acid). The reader was previously calibrated against a phosphate standard curve (0–8.27 µg/mL). Readings were performed using the absorbance of λ 660 nm^32^. The ionic concentration of phosphate was obtained from the absorbance values using a linear equation (y = ax + b) in a curve fit above 0.99. Samples were analyzed in duplicate. Phosphate concentrations were expressed as µg/mL.

### Fluoride analysis

Fluoride concentration in saliva was measured by the direct method^33^. A specific electrode for fluoride ion (Thermo Scientific Orion Eletrode—Model 9409BN, Fisher Scientific, Cambridge, MA, USA) and a potentiometer (Thermo Scientific Orion—Model 720, Fisher Scientific, Cambridge, MA, USA) were used for fluoride analysis and each sample was analyzed in duplicate. Before the readings, calibration curves were obtained with fluoride standards ranging from 0.01 to 0.1 mg F/mL and TISAB III (1:10) (TISAB III Concentrate for Fluoride Electrode, Fisherbrand, Walthamam, MA, USA).

Results expressed in mV by the potentiometer were converted to concentration of fluoride ion using a standard correlation curve (r^2^ > 0.99) based on the linear regression of the calibration curve. Fluoride concentrations were expressed as µg/mL.

### Statistical approach

Statistical Package for the Social Sciences software (IBM SPSS Statistics for Windows—version 21.0, Armonk, NY, USA) and GraphPad Prism software (GraphPad Software for Windows—version 7.04, San Diego, California USA) were used for statistical inferences.

The normality of data variances was checked using the Shapiro–Wilk test. Data of calcium, phosphate, and fluoride followed the Gaussian distribution and the equality of variances. Data of caries index were represented as a median and interquartile range due to non-Gaussian distribution.

The three-way mixed model of analysis of variance was used to determine the effect of biofilm accumulation and sucrose rinse (within subjects’ effects) and early childhood caries (between subjects’ effects) on the test variables (dependent variables: Ca^2+^, P_i_, and F^−^). The Box's M test was used to prove the equality of multiple variance–covariance matrices considering the 0.001 significance level. Significant interaction in analysis of variance ruled the need for simple effects tests to reveal the degree to which one factor is different from other factors, considering the Bonferroni adjustment to avoid familywise error. The level of significance established for the analysis was 0.05, considering the two-tailed test hypothesis.

### Ethics declarations

Approval for human experiments. Methods were carried out following the Helsinki declaration and the regulatory guidelines and norms obey the 466/12 resolution for research ethics in Brazil. The Research Ethics Committee of the Piracicaba Dental School University of Campinas approved this research. (CAAE: 70777517.9.0000.5418). Parents or guardians who agreed to the inclusion of their children in the research, filled out an informed consent form (FICF), authorizing their participation.
